# Supplementary material for: A Head-to-Head Comparative Study of the Replication-Competent Vaccinia Virus and AAV1-Based Malaria Vaccine versus RTS,S/AS01 in Murine Models
Source: Vaccines (Basel). 2024 Oct 10;12(10):1155. doi: 10.3390/vaccines12101155 (PMC11512279; doi:10.3390/vaccines12101155)
Supplement: Supplementary file 1 [file vaccines-12-01155-s001.zip › vaccines-3238088-supplementary.pdf]

vaccines

MDPI

Supplementary Figures

Figure S1

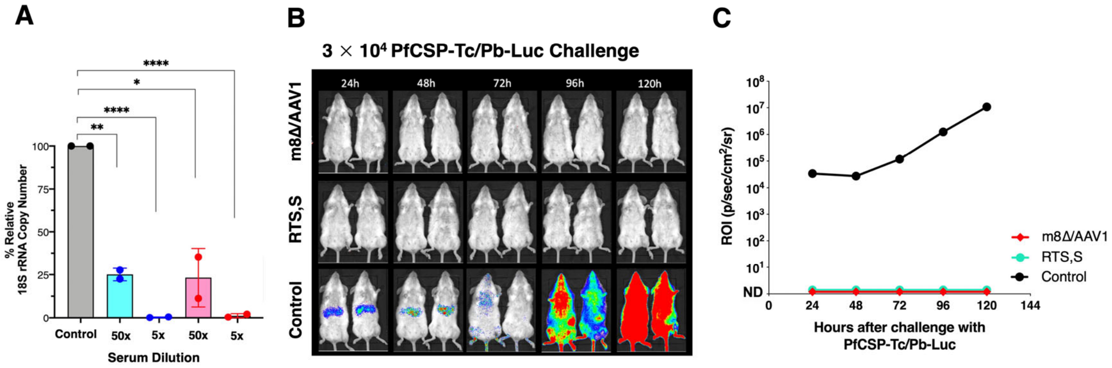

Figure S1. Transgenic sporozoite-neutralizing activity. **(A)** Sera from BALB/c mice immunized with m8Δ/AAV1 or RTS,S/AS01 (RTS,S) were tested in transgenic sporozoite neutralization assay. PfCSP-Tc/Pb sporozoites were incubated with control mice sera or immune sera at two serum dilutions (1:5 or 1:50) before addition to HepG2 cells. After 48 h of culture, levels of parasites in HepG2 cell extracts were measured by real-time PCR. Results are shown as percentage relative 18S rRNA copy number. Differences between groups were analyzed using an unpaired *t*-test. **p* < 0.05, ***p* < 0.01, *****p* < 0.0001. **(B)** BALB/c mice, *n* = 2 per group, were inoculated with m8Δ/AAV1 or RTS,S to form the immunized groups, and PBS was used for the control group. Four weeks after the final immunization, mice were challenged with 3 × 10^4 PfCSP-Tc/Pb sporozoites expressing the luciferase gene (PfCSP-Tc/Pb Luc). The mean total photon flux (whole body) from 24–120 h after intravenous administration of PfCSP-Tc/Pb Luc is shown. ROI: region of interest, ND: not detected.

Figure S2

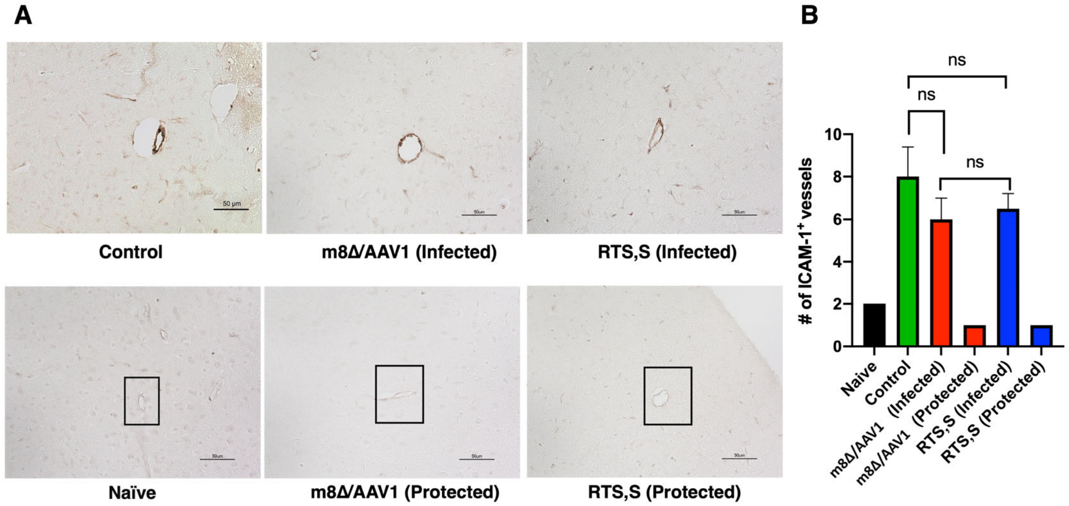

Figure S2. Assessment of vaccine efficacy against cerebral malaria. Immunohistochemical staining for ICAM-1 in cerebral vessel endothelia of vaccinated C57BL/6 mice on day 8 after challenge with 1 × 10^3 Pfs25-PfCSP/Pb sporozoites. **(A)** Representative images showing ICAM-1 expression in control, m8Δ/AAV1 (infected), RTS,S (infected) and almost no ICAM-1 expression in naïve, m8Δ/AAV1 (protected), and RTS,S (protected) groups. Scale bar: 50 μm. **(B)** Quantification of ICAM-1-positive vessels across different groups. Data represent the mean number of positive vessels per 20 fields at 400× magnification for each group; error bars denote SEM. Differences between groups were analyzed using unpaired *t*-test. "ns" indicates no significant differences between groups.
